# Human African Trypanosomiasis in the Democratic Republic of the Congo: A Looming Emergency?

**DOI:** 10.1371/journal.pntd.0001950

**Published:** 2012-12-13

**Authors:** Epco Hasker, Pascal Lutumba, François Chappuis, Victor Kande, Julien Potet, Anja De Weggheleire, Charles Kambo, Evelyn Depoortere, Bernard Pécoul, Marleen Boelaert

**Affiliations:** 1 Institute of Tropical Medicine, Antwerp, Belgium; 2 University of Kinshasa, Kinshasa, Democratic Republic of the Congo; 3 Division of International and Humanitarian Medicine, Geneva University Hospitals & University of Geneva, Geneva, Switzerland; 4 Programme National de Lutte contre la Trypanosomiase Humaine Africaine, Kinshasa, Democratic Republic of Congo; 5 Médecins Sans Frontières - Access Campaign, Paris, France; 6 Médecins Sans Frontières, Kinshasa, Democratic Republic of the Congo; 7 CDI Bwamanda, Democratic Republic of the Congo; 8 Drugs for Neglected Diseases initiative, Geneva, Switzerland; Foundation for Innovative New Diagnostics (FIND), Switzerland

Human African trypanosomiasis (HAT), also known as “sleeping sickness”, is a parasitic disease that is fatal if left untreated. The disease is transmitted by the tsetse fly and is endemic to sub-Saharan Africa, where it mainly affects impoverished rural communities. There are two forms of HAT; the West African variant caused by *Trypanosoma brucei gambiense* is the most common and accounts for over 90% of the current case load. The disease occurs in two stages, the haemato-lymphatic stage (stage 1) with no or few specific symptoms, followed by the meningo-encephalitic stage (stage 2), which occurs when the parasite has crossed the blood-brain barrier. This second stage is characterized by neurological signs and personality changes; damage to the hypothalamus can lead to disturbances of the normal sleep/wake cycle, hence the name “sleeping sickness” [1].

The ominous reputation of HAT is linked to a history of devastating epidemics, controlled with sometimes draconian measures, only to re-emerge again once control measures were abandoned. At the turn of the 20^th^ century, an estimated 300,000 to 500,000 people died from HAT; another major epidemic occurred in the 1920s–'30s [2]. By the mid-1960s, the disease was almost eliminated, but 30 years later incidence levels were back to where they had been in the 1920s [3]. This is illustrated by data from the Democratic Republic of the Congo (DRC), the worst affected country (Figure 1). After independence in 1960, the strict and costly HAT control measures put in place by the colonial authorities were relaxed and the number of HAT cases gradually increased. Operations of the national sleeping sickness control programme (Programme National de Lutte contre la Trypanosomiase Humaine Africaine, PNLTHA) continued to be funded mainly by the Belgian government. When in 1991 all Belgian bilateral aid, including the support to the PNLTHA, was suspended because of international sanctions against the Mobutu regime, the epidemiological situation soon became dramatic.

**Figure 1:**
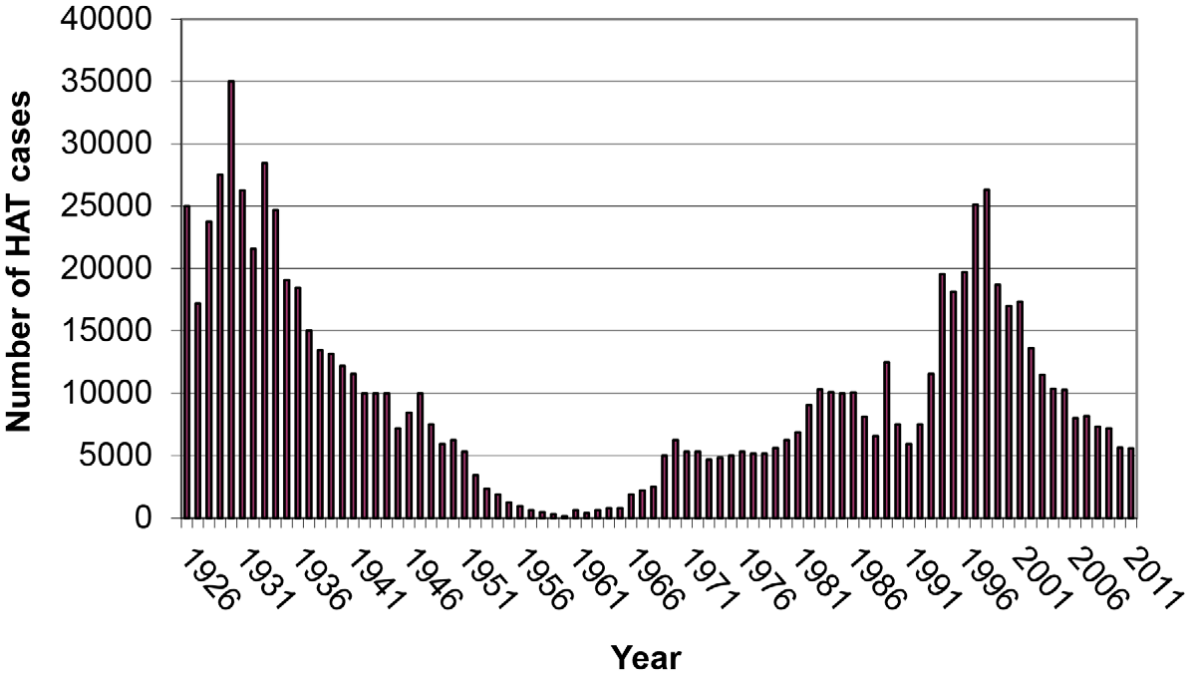
Annual case notification of HAT in DRC, 1926–2011. Source: PNLTHA.

In 1998, a total of 40,000 cases were reported worldwide, 66% of which (26,318 cases) in the DRC. The WHO estimates that 300,000 cases remained undetected and therefore untreated [4]. An emergency HAT control program was launched and followed up by the current program, which is largely funded through bilateral cooperation with Belgium. Active screening was resumed, reaching up to 3 million persons per year, at an annual cost of 2.8 million US$. However, in the perspective of this external funding being phased out, and with no alternative funding sources identified as of yet, the PNLTHA has been forced to gradually scale down active case finding campaigns.

In 2009, *T.b. gambiense* HAT was still focally endemic in 24 Sub-Saharan African countries, but out of the 9,688 cases reported globally, 7,326 (76%) occurred in the DRC [5]. The provinces of Bandundu and Kasai Oriental remain heavily affected; the situation in Province Orientale is unclear, because on-going armed conflict interferes with screening campaigns, but more than 3,000 cases have been diagnosed over the last 5 years [6].

## Averting a Looming Emergency

The recent evolution of HAT control activities in the DRC calls for urgent action to prevent a new resurgence. Similar considerations apply to the other countries where HAT is endemic.

### Continuity of Control Efforts

Because humans are assumed to be the sole reservoir for *T.b.gambiense* HAT, active population screening combined with treatment of cases has since long been the backbone of HAT control. When control efforts are scaled down in response to low prevalence, increasing numbers of infected persons are not identified and do not receive treatment, making transmission of the parasite possible [7]. It is only a matter of time before prevalence goes up again to reach epidemic levels [8]. This is exactly what is at risk of happening in the DRC at the moment. Control efforts of the past decade have paid off, and disease transmission is under control in most, but certainly not in all provinces. Abandoning control measures at this stage, while transmission is still on-going, guarantees a progressive increase of prevalence in the coming years.

It would be a wasteful use of resources to let that happen. The investments made in the past decade by the Congolese national control program, building the expertise to ensure the complex screening, diagnostic and curative activities, in addition to ensuring the demanding logistic support, all risk being lost. In the provinces of Bandundu, Kasai Oriental and Orientale, the disease is not under control yet and reinforcement of the control measures is needed. In order to avoid re-emergence of HAT in other areas, the available expertise needs to be maintained and mobilized in the most appropriate way.

### Integration of HAT Control

In a context of substantial decrease of case notifications and reduced budgets for active screening, integration of HAT control into general primary healthcare seems a logical next step. Efforts to integrate HAT control services have been on-going since 2008, but a number of issues related to the disease and to the health system represent serious constraints to this integration effort.

To date there is no simple diagnostic test available that can be applied routinely in a rural health centre in sub-Saharan Africa. HAT diagnosis usually involves a two-step procedure, using the Card Agglutination Test for Trypanosomiasis (CATT), followed by a parasitological confirmation test [9], [10]. The CATT test is easy to perform but requires a battery-powered rotator, as well as a cold chain; parasitological confirmation tests require at least a functional microscope. Because the treatment regimen depends on disease stage, a lumbar puncture for stage determination is necessary. Current treatment regimens for HAT are complex, requiring one week of daily intramuscular injections for patients in stage 1 or twice daily intravenous perfusions for patients in stage 2.

The local health system is ill equipped to deal with a rare disease with a non-specific early clinical presentation that requires complex diagnostics and treatment. Passive case finding has been shown to entail long delays and missed diagnoses [11], [12]. The mean duration of stage 1 and 2 is estimated at 526 and 500 days, respectively [13]. Since most patients detected passively are diagnosed only when in stage 2, they have been infectious to the vector (and therefore sustaining transmission) for at least one or two years. Moreover, patients diagnosed in stage 2 are at higher risk for irreversible sequelæ and require a more complex treatment [14]. Despite efforts aimed at integration, the proportion of cases detected through passive screening in the DRC have remained relatively stable over the past four years, apparently not compensating for the decrease in active screening.

Even if problems related to diagnostic and treatment procedures could be overcome, one more constraint to the integration of HAT control activities remains. In the DRC, the average annual attendance rate of health services is estimated at 0.15 per inhabitant [15]. Unless a significant effort is made to further develop health services and increase their utilization, it is an illusion to think the current health system is sufficient to control HAT or to detect resurgence at an early stage. Until then, some form of active surveillance will continue to be required.

### Control in Low Prevalence Contexts

Once prevalence levels go down, populations in HAT endemic areas no longer perceive the disease as a threat. Under such circumstances, people are no longer prepared to participate in the screening campaigns because these are time consuming, have little respect for privacy, and because people fear the side effects of HAT treatment [16]. In addition, in a low prevalence context, screening activities become less efficient. The province of Equateur in the DRC was the focus of a major epidemic in the 1990s. In 2011, only 219 HAT cases were diagnosed in this province, of which 62 by mobile screening teams; yet during that year, 5 mobile screening units made up of 33 staff members in total, had screened 144,281 persons (personal communication Dr. Christian Roberti).

A poor yield should not be a pretext for abandoning active screening activities, but rather serve as a call for innovative screening methods to be developed and field tested. Laveissière described in 1998 how health workers going from house to house collecting blood samples on filter papers that were later processed in laboratories, were able to reach more people at a lower cost when compared to mobile teams [17]. Difficulties in communicating test results and in tracing back HAT suspects have until now been the main reasons for keeping with the mobile team approach. Nowadays, modern communication technology has penetrated the most distant corners of rural Africa and geographic positioning systems (GPS) that were once unaffordable can be bought for less than 100 USD. Using a rapid test currently undergoing phase III evaluation would allow the immediate communication of the screening test result, while still in the village. A Loop-mediated isothermal Amplification (LAMP) test that can provide direct evidence of the presence of *T.b. gambiense* DNA in a sample is currently under development [18]. This test can be performed on samples collected on filter paper and can be carried out with equipment that is automatized to such a degree that it can be used at the level of a district laboratory. If the diagnostic accuracy of this new test can be confirmed, the combination of rapid tests and LAMP with the use of GPS technology would make it possible to locate and map all those probably infected with *T.b. gambiense* in a very efficient manner. Those individuals could then be invited for a further diagnostic workup in the privacy of a consultation room in a larger health center or district hospital, where they could also be started on treatment.

A new treatment regimen based on 10 days of once daily oral administration of fexinidazole is currently entering phase II clinical trials. If these trials are successful, fexinidazole could allow the diagnostic threshold to be lowered, because it is easy to administer and expected to be less toxic. This drug passes the blood brain barrier and would be suitable for both stage 1 and stage 2 patients, thus obviating the need for a lumbar puncture [19].

## Conclusion

After a major upsurge in the 1990s, HAT is now under control in most endemic regions. To prevent resurgence from re-occurring, which we know will happen if control efforts are relaxed, the continuation of control activities is crucial. In the DRC the knowledge, expertise and capacity are in place; continued funding of the national HAT control programme would allow for these to be utilized and the epidemiological situation to be kept under control.

With new, easy to perform diagnostic tests and an oral treatment regimen in the pipeline, integration of HAT control into general healthcare services can be considered. However, integration will not be sufficient to prevent HAT resurgence in the longer term. Continued strong surveillance, covering at least the historically known HAT foci, is crucial, and cannot rely on passive case finding alone. At the same time, under the current circumstances of lowering prevalence, active surveillance using mobile teams is neither effective nor sustainable. Possible alternative approaches to surveillance have been identified but still need to be field tested and optimized.

HAT is listed by the WHO as one of the diseases requiring “Innovative and Intensified Disease Management” [20]. In the coming years, a gradual and well-managed integration of the HAT control activities in the general health services can be envisaged, but this will require further research to identify the best strategies in low prevalence contexts. A minimal vertical approach to control the disease is likely to remain necessary. Finally, a regional control strategy, coordinated between countries where HAT is prevalent, should be actively explored.
